# *In Vivo* Corneal Biomechanical Properties with Corneal Visualization Scheimpflug Technology in Chinese Population

**DOI:** 10.1155/2016/7840284

**Published:** 2016-07-14

**Authors:** Ying Wu, Lei Tian, Yi-fei Huang

**Affiliations:** ^1^Department of Ophthalmology, Chinese PLA General Hospital, Beijing 100853, China; ^2^Beijing Institute of Ophthalmology, Beijing Tongren Eye Center, Beijing Tongren Hospital, Capital Medical University, Beijing Ophthalmology & Visual Sciences Key Laboratory, Beijing 100730, China

## Abstract

*Purpose*. To determine the repeatability of recalculated corneal visualization Scheimpflug technology (CorVis ST) parameters and to study the variation of biomechanical properties and their association with demographic and ocular characteristics.* Methods*. A total of 783 healthy subjects were included in this study. Comprehensive ophthalmological examinations were conducted. The repeatability of the recalculated biomechanical parameters with 90 subjects was assessed by the coefficient of variation (CV) and intraclass correlation coefficient (ICC). Univariate and multivariate linear regression models were used to identify demographic and ocular factors.* Results*. The repeatability of the central corneal thickness (CCT), deformation amplitude (DA), and first/second applanation time (A1/A2-time) exhibited excellent repeatability (CV% ≤ 3.312% and ICC ≥ 0.929 for all measurements). The velocity in/out (*V*_in/out_), highest concavity- (HC-) radius, peak distance (PD), and DA showed a normal distribution. Univariate linear regression showed a statistically significant correlation between *V*_in_, *V*_out_, DA, PD, and HC-radius and IOP, CCT, and corneal volume, respectively. Multivariate analysis showed that IOP and CCT were negatively correlated with *V*_in_, DA, and PD, while there was a positive correlation between *V*_out_ and HC-radius.* Conclusion*. The ICCs of the recalculated parameters, CCT, DA, A1-time, and A2-time, exhibited excellent repeatability. IOP, CCT, and corneal volume significantly influenced the biomechanical properties of the eye.

## 1. Introduction

The cornea acts as the outermost lens of the eye and aids in the focus and transmission of light. It provides mechanical stability to the eye and acts as a protective layer to the interior of the eye. Eye disorders, surgeries, and injuries may alter the shape, thickness, or biomechanical properties of the corneas, resulting in serious changes in visual performance of the eye [1–3]. Clinically, it is very extremely important to understand the biomechanical responses of cornea toward intraocular pressure (IOP), refractive surgery, and corneal pathology. To study the biomechanics of cornea, various methods have been devised [4–6]. One such method is the* in vivo* measurement of the biomechanical responses of the cornea using an ocular response analyzer (ORA; Reichert, Buffalo, NY, USA), one of the earliest commercially available devices [7]. Several studies have been published with diverse and new data regarding corneal hysteresis (CH) and corneal resistance factor (CRF) in healthy and pathologically challenged eyes [8, 9]. However, ORA, which reports nonstandard biomechanical terms that are different from the classical knowledge of corneal biomechanics, does not provide information associated with direct corneal deformation and cannot exhibit the corneal dynamic deformation process in real-time [10].

Recently, to overcome the lack of more direct measurement techniques of corneal deformation* in vivo*, a number of new approaches have been attempted, although these techniques are still in their early stages. A new device, corneal visualization Scheimpflug technology (CorVis ST; Oculus Optikgeräte GmbH, Wetzlar, Germany), designed to measure corneal deformation characteristics with a high-speed Scheimpflug camera, has been introduced. Measurement of corneal biomechanical properties using CorVis ST has received increasing attention recently [11, 12]. Biomechanical parameters may be associated with diseases such as keratoconus, stromal keratopathy, and glaucoma, which may be measured to evaluate the effect of corneal refractive surgery and corneal cross-linking [13–17]. Leung et al. [18] reported that the influence of deformation amplitude (DA) on the measured error of Goldmann applanation tonometry is more in comparison to the central corneal thickness (CCT). Therefore, independent studies should focus on determining “normal” values in different populations, so that the new technology identifies wider acceptance and extensive use at clinical levels. The present study aimed to justify the repeatability of recalculated CorVis ST parameters and the variation of biomechanical properties provided by CorVis ST in a healthy Chinese population and their association with demographic and ocular characteristics.

## 2. Methods

### 2.1. Subject Recruitment

A total of 783 healthy Chinese subjects, aged 13–89 years, were recruited between July 2013 and August 2015 at the Chinese General Hospital of the People's Liberation Army (PLA, Beijing, China). This cross-sectional study was approved by the office of Research Ethics Committee of the Chinese PLA General Hospital in accordance with the tenets of the Declaration of Helsinki. Written informed consent was provided by all the subjects prior to entering the study.

### 2.2. Ocular Examinations

All the participants underwent a comprehensive ophthalmological examination and a standardized interview procedure. The ocular examinations included a detailed assessment of visual acuity, slit-lamp microscopy and fundus examination, corneal tomography (Pentacam; Oculus Optikgeräte GmbH, Wetzlar, Germany), corneal biomechanics, and IOP measurement (CorVis ST). All the assessments were performed at the same time during a single visit, to decrease the effect of diurnal variations.

The exclusion criteria for the subjects were as follows: any previous corneal or ocular surgery, any ocular pathology or systemic diseases known to affect the eye, or chronic use of topical medications.

### 2.3. CorVis ST Measurement

**Figure 1:**
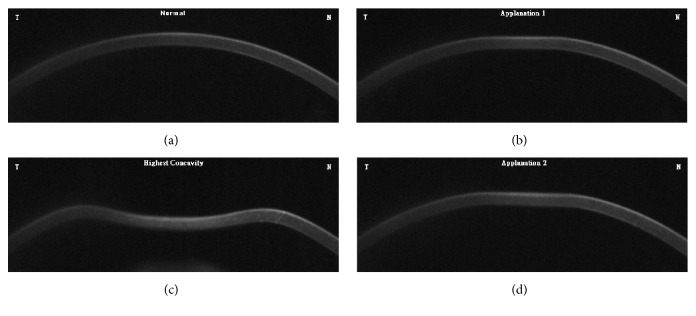
Deformation process observed using CorVis ST. (a) The recording starts with the cornea in a natural convex shape. (b) A precisely metered air pulse forces the cornea to move inward through an applanation (1st applanation). (c) The cornea then continues to move inward until it reaches the highest concavity. (d) The cornea rebounds to its normal convex shape. During this phase, the cornea again passes through an applanation (2nd applanation).

The CorVis ST instrument is a noncontact tonometer and imaging device that provides additional information regarding the reaction of cornea to a defined air pulse. It is mainly used to measure IOP and CCT. An ultrahigh-speed Scheimpflug camera (which can record 4,330 frames/sec) was used to capture the corneal deformation with a corneal horizontal range of 8.5 mm. The video clip containing 140 digital frames corresponded to a recording time of ~30 msec (Figure 1). In brief, the process was as follows: first, an image of the cornea was recorded by the ultrahigh-speed Scheimpflug camera, prior to the air puff in the natural convex shape. Subsequently, the cornea was made to move inward and flatten to the first applanation (A1), by precisely applying a metered air pulse to the cornea. The cornea continued to move inward until it reached a position of highest concavity (HC). As cornea is viscoelastic, it rebounded from this concavity to the second applanation (A2) and subsequently to its normal convex curvature. The IOP was determined according to Imbert-Fick's law based on A1-time [19]. The pachymetric values were detected when the cornea was in its natural shape and when there was no air pulse [19, 20]. Abbreviations shows the 10 biomechanical parameters measured by CorVis ST. The latest version of the software by Oculus (version 6.07r24) was used to recalculate all the CorVis ST measurements, which assisted in obtaining more precise data associated with the described parameters. For example, HC-radius presented an improvement from a three-point fit calculation obtained from previous software versions (version 1.0r30) to a new parabolic fit computation [21]. The quality-specification section on the output map was used to check the quality of the examination. A reading with an “OK” was considered acceptable; otherwise, the measurements were repeated.

The CorVis ST is similar to the dynamic bidirectional applanator in that it uses an air puff to deform the cornea and records the process by a Scheimpflug camera. From the real-time series of images, deformation characteristics can be extracted along with the potential to quantify corneal elastic parameters. For example, the slower movement in *V*_in_ and *V*_out_ is associated with a stiffer cornea, since it takes greater force to deform a stiffer material and reach the same velocity. For the parameter of DA, it has been hypothesized that this may correspond to the stiffness of the cornea since a softer cornea with a lower elastic modulus would have greater deformation under the same load. Therefore, softer corneas would produce deep, narrow deformations, and stiffer corneas would produce wide, shallow deformations [22]. The A2-time may represent the time-dependent portion of the viscoelastic response and can be considered as an indication of the overall viscoelasticity [23].

Previous studies have reported that the IOP measurements using CorVis ST and Goldmann applanation tonometry showed satisfactory levels of agreement [24, 25]. Therefore, in the present study, the IOP reading provided by CorVis ST was used for data analysis.

### 2.4. Statistical Analysis

Two software programs, SPSS version 17.0 (SPSS, Inc., Chicago, IL, USA) and MedCalc 13.0 (MedCalc Software, Ostend, Belgium), were used to conduct the statistical analyses. Kolmogorov-Smirnov test was used to estimate the normality of distribution of the measured variables. Welch's modified Student's two-sample *t*-test and the Wilcoxon rank-sum test were used to determine differences in biomechanical parameters among gender groups. Linear regression analysis was performed to assess the effect of each variable on *V*_in_, *V*_out_, DA, PD, and HC-radius and multivariate linear regression models were further conducted with *V*_in_, *V*_out_, DA, PD, and HC-radius as the dependent variables and age, gender, IOP, CCT, mean keratometry (*K*_*m*_), and anterior chamber depth (ACD) as covariates. A *P* value < 0.05 was considered to indicate a statistically significant difference. Repeatability analysis was carried out on the recalculated biomechanical parameters (obtained using a new version of the software). The analysis was conducted by measuring three measurements accomplished by a single operator, on 90 eyes of the study samples. Precision, repeatability, coefficient of variation, and intraclass correlation coefficients (ICCs) were the factors calculated for the parameter repeatability analysis [26]. ICC was interpreted as follows: <0.75, poor to moderate repeatability; 0.75–0.90, good measurement repeatability; and >0.90, excellent repeatability for clinical measures [27].

## 3. Results

### 3.1. Repeatability

The repeatability of the recalculated biomechanical parameters was measured through 3 measurements (taken in a gap of 3 min on the same day) and was conducted on 90 eyes of the study samples.  Table 1 shows the precision, repeatability, coefficient of variation, and ICC of the parameters. CCT, DA, A1-time, and A2-time showed excellent repeatability, with a CV% of 0.925%, 3.312%, 1.306%, and 0.657% and ICC of 0.983, 0.942, 0.929, and 0.929, respectively. PD, IOP, and *V*_out_ showed good repeatability, with a CV% of 1.701%, 6.245%, and −9.834% and ICC of 0.896, 0.894, and 0.768, respectively. By contrast, ICCs of the other parameters were <0.75 with a moderate or poor level.

### 3.2. Characteristics of Healthy Subjects

**Figure 2:**
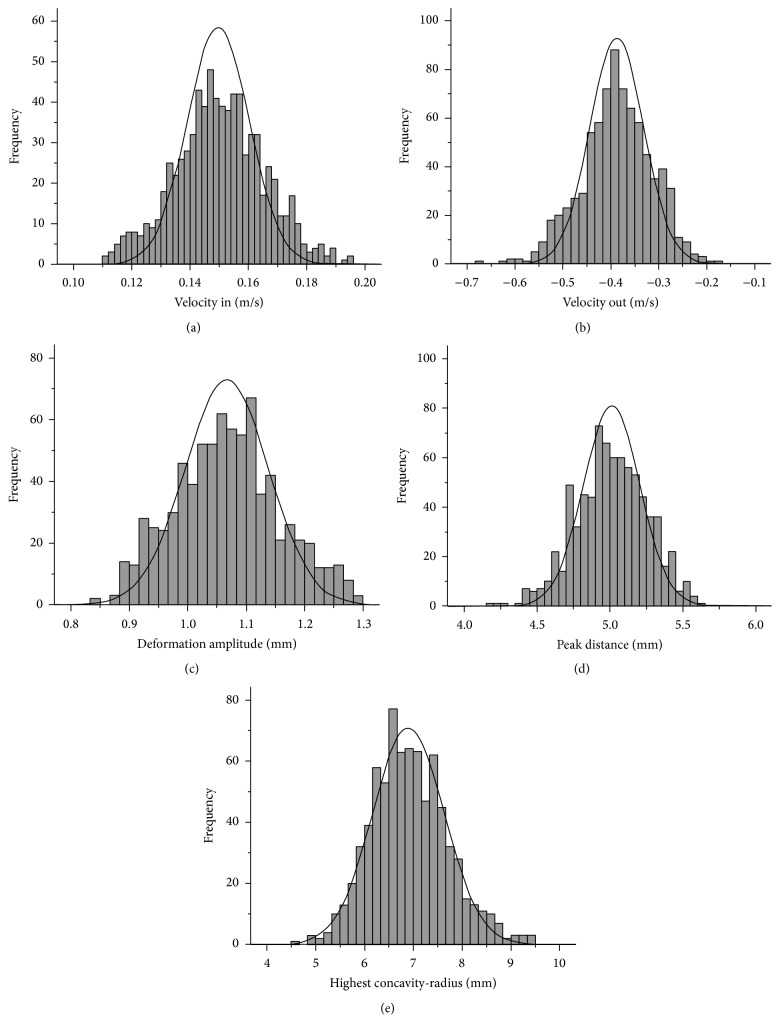
Distribution of corneal biomechanical parameters in normal Chinese population. (a) Velocity in; (b) velocity out; (c) deformation amplitude; (d) peak distance; (e) highest concavity-radius.

A total of 783 healthy Chinese subjects (402 females, 51.34%; 381 males, 48.66%), with a mean age of 34.93 ± 17.65 years, were recruited in this study. The mean IOP and astigmatism of eyes were 14.14 ± 2.19 mmHg and 1.08 ± 0.7 diopters, respectively. The mean CCT was 541.14 ± 32.67 *μ*m, mean keratometry was 43.63 ± 1.45 diopters, and mean corneal volume was 60.1 ± 3.69 mm^3^. The mean anterior chamber angle, depth, and volume were 37.03 ± 7.58 degrees, 3.08 ± 0.45 mm, and 175.14 ± 43.98 mm^3^, respectively. Kolmogorov-Smirnov test confirmed the normality of the biomechanical parameter distributions. The parameters, such as *V*_in_, *V*_out_, HC-radius, PD, and DA, showed a normal distribution (Figure 2).  Table 2 showed that there was no significant difference between male and female subjects with respect to the 10 biomechanical parameters observed in the study (*P* > 0.05).

### 3.3. Determinants of Corneal Biomechanical Parameters

Table 3 showed the investigative results of the univariate linear regression analysis and multivariate models. *V*_in_, *V*_out_, DA, PD, and HC-radius showed no significant relationship with age and gender, whereas all of them varied in terms of IOP, CCT, and corneal volume.

## 4. Discussion

The present study was one of the first studies that explored the correlation between gender variations and corneal biomechanical properties (provided by CorVis ST) and also their association with several demographic and ocular factors in a Chinese population. As knowledge regarding the corneal biomechanical properties and the influence of demographic and ocular characteristics on the corneal deformation response aid in predicting and diagnosing certain biomechanics-related ocular diseases, such as keratoconus during clinical practice, it is believed that study of these factors is important [11, 28, 29]. CorVis ST displays information on corneal deformation in real-time and allows a direct description of the mechanical behavior of the cornea [30]. The biomechanical parameters provided by CorVis ST may be associated with diseases such as keratoconus [11], glaucoma [15], and diabetes mellitus [31].

This study has shown that CCT, DA, A1-time, and A2-time exhibit excellent repeatability, followed by PD, IOP, and *V*_out_, which exhibited good repeatability levels. The biomechanical parameters, *V*_in_, *V*_out_, DA, PD, and HC-radius, showed a normal distribution in the healthy Chinese population. No significant differences were identified between male and female subjects in terms of the 10 biomechanical parameters evaluated. Univariate linear regression showed that *V*_in_, *V*_out_, DA, PD, and HC-radius showed statistically significant correlations with IOP, CCT, and corneal volume, respectively. It was found that IOP and CCT were still negatively correlated with *V*_in_ and also with DA and PD, even following adjustment for age, gender, IOP, CCT, *K*_*m*_, and ACD, whereas they were positively associated with *V*_out_ and HC-radius.

In a repeatability study of CorVis ST measurements, Hon and Lam [30] reported that CCT was the most repeatable corneal parameter measured by this device, followed by DA and A1-time, which was consistent with the results of Ali et al. [28]. In the present study, CCT, DA, A1-time, and A2-time showed excellent repeatability for the recalculated parameters. Previous studies had limitations of the software version, due to which the repeatability of PD and HC-radius had not been tested [18, 20]. In the present study, the ICCs were 0.896 and 0.605 for the recalculated PD and HC-radius, respectively. With the use of the new software version, the repeatability of the recalculated biomechanical parameters showed an improvement.

In the present study sample of Chinese subjects, the mean *V*_in_, *V*_out_, DA, PD, and HC-radius were 0.15 ± 0.02 m/s, −0.39 ± 0.07 m/s, 1.07 ± 0.09 mm, 5.01 ± 0.24 mm, and 6.93 ± 0.79 mm, respectively. Similar mean values of DA have been reported in studies that investigated diverse ethnicities (DA, 1.07 ± 0.10 mm) [28] and Brazilian subjects (DA, 1.05 ± 0.08 mm) [32].

In the present study, no significant correlations were identified between age and biomechanical parameters measured by CorVis ST. By contrast, certain previous studies have reported that structural changes in human corneal stroma are age-related, as they present an increase in stromal fibril diameter and interfibrillar cross-linking, contributing to increased stiffness [33, 34]. In addition, Elsheikh et al. [35] reported a similar correlation between age and corneal structure. Their study experimentally demonstrated that the cornea considerably stiffened with age, with a linearly related and increased Young's modulus of elasticity. As stiffness of the cornea increases with age, an older cornea would probably yield a lower DA and higher HC-radius. The findings of the present study appear to contradict these evidences, as this study showed no correlations between age and corneal biomechanical parameters. Nemeth et al. [20] reported that the 10 specific CorVis ST parameters showed no significant association with age. Similar findings have been reported by Hon and Lam [30], which suggested that the biomechanical parameters were not correlated with age and only HC-time showed a weak positive correlation (*r* = 0.18, *P* = 0.04) in a group of healthy subjects from a Brazilian population. Upon reviewing the aforementioned parameters, it was found that the CorVis ST instrument may not be sensitive enough to detect the change in biomechanical properties that is associated with age difference.

Hon and Lam [30] found that the biomechanical parameters were not associated with gender and there was no significant difference in the parameters between female and male subjects.

A positive correlation of *K*_*m*_ was identified with *V*_in_, and a negative correlation was found with PD in univariate linear regression models; however, *K*_*m*_ had no significant correlation with *V*_out_, DA, and HC-radius. These results were confirmed by earlier studies, as they also reported that DA was not correlated with corneal curvature [18, 29].

Until now, there have been no studies on the association of corneal volume, ACD, and ACV with biomechanical parameters measured using CorVis ST. However, it was found that corneal volume was negatively correlated with *V*_in_, DA, and PD but positively correlated with *V*_out_ and HC-radius. Several studies have shown that, compared to normal controls, the values for corneal volume and CCT are significantly lower in keratoconus eyes [36, 37]. Mannion et al. [38] identified that when there is loss of corneal tissue, particularly in the central and paracentral area, a significant decrease in corneal volume in keratoconus is indicated. The decreases in corneal stiffness and reduction in corneal volume may be associated with each other. ACD and ACV were found to have a negative correlation with *V*_out_ and HC-radius and a positive correlation with PD. However, further studies are warranted to understand the role of anterior chamber parameters and their association with the corneal deformation parameters in an improved manner.

Several previous studies have reported that CCT is negatively correlated with DA [18, 29, 39]. The present study also showed similar results. In a study conducted by Hon and Lam [30], it was reported that CCT was negatively correlated with *V*_in_ and positively correlated with *V*_out_ and HC-radius. This type of correlation may be due to the fact that the stiffness and elasticity are directly proportional to the corneal thickness, showing that as the corneal thickness increases, the other two mentioned parameters also increase. The effectiveness of corneal collagen fibers, the main contributors to corneal stiffness, may be less in those subjects having less CCT.

IOP is an important factor that affects the value of the biomechanical parameters. A clinical prospective observational case-control study reported that IOP has significant correlation with *V*_in_, *V*_out_, DA, and HC-radius, which shows that it has an important influence on corneal biomechanical behavior [39]. Furthermore, an* ex vivo* study using porcine cornea [40] and certain contact lens corneal models also verified the strong influence of IOP on corneal deformation [41]. In the present study, IOP presented a significant negative correlation with *V*_in_, DA, and PD and significant positive correlation with *V*_out_ and HC-radius, which indicated that greater IOP causes a higher pressure threshold for the cornea to move, leading to lower *V*_in_. In addition, DA is lower at higher IOP; hence the cornea rebounds sooner, leading to greater *V*_out_ in the recovery phase. Elsheikh et al. [42] measured the stiffness of 37 corneas from human donors using inflation tests and demonstrated that there was a positive linear association between Young's modulus and IOP. This result indicates that the cornea is less likely to deform when IOP is high. Therefore, IOP should be taken into account while conducting comparisons between the study populations as it has an important role in corneal biomechanical behavior by influencing the corneal deformation response.

The coefficients of determination (the *R* ^2^ value) in multiple linear regressions of *V*_in_, *V*_out_, DA, PD, and HC-radius were 0.205, 0.341, 0.298, 0.425, and 0.196, respectively. This implies that the variations in the IOP and anterior segment parameters explain 20–40% of the variance of the corneal biomechanical properties and the innate corneal properties explain the remaining 60–80% variance.

However, the present study has a few limitations. First, this was an observational cross-sectional study, which may limit causal inferences; and second, as this study focused only on Chinese population, it is not known whether these results can be extrapolated to other ethnicities.

In conclusion, to the best of our knowledge, this was the first study to profile variations in corneal biomechanical properties measured by CorVis ST in a large, unselected Chinese population. IOP, CCT, and corneal volume significantly influenced the biomechanical properties of the eye. These results may be relevant while investigating the role of altered corneal biomechanics in ocular diseases, such as corneal degeneration and glaucoma. As the CorVis ST is a relatively new technology, more studies should be conducted on the applicability and capabilities of this imaging technique for characterizing corneal biomechanics.

## Figures and Tables

**Table 1:** Repeatability of the recalculated corneal visualization Scheimpflug technology parameters for three measurements^a^.

Parameters	Precision	Repeatability	CV%	ICC	95% CI for ICC
First applanation time (msec)	0.187	0.264	1.306	0.929	0.901~0.950
First applanation length (mm)	0.113	0.160	3.221	0.223	0.094~0.362
Velocity in (m/sec)	0.018	0.026	6.353	0.695	0.601~0.776
Second applanation time (msec)	0.284	0.401	0.657	0.929	0.901~0.951
Second applanation length (mm)	0.540	0.764	16.153	0.230	0.101~0.369
Velocity out (m/sec)	0.076	0.107	−9.834	0.768	0.690~0.832
Highest concavity time (msec)	0.684	0.966	2.093	0.302	0.171~0.438
Deformation amplitude (mm)	0.069	0.098	3.312	0.942	0.920~0.960
Peak distance (mm)	0.168	0.238	1.701	0.896	0.857~0.927
Highest concavity radius (mm)	1.136	1.606	8.320	0.605	0.495~0.704
Intraocular pressure (mmHg)	1.696	2.397	6.245	0.894	0.854~0.925
Central corneal thickness (*μ*m)	9.642	13.627	0.925	0.983	0.976~0.988

^a^ *n* = 90. CV: coefficient of variation; ICC: intraclass correlation coefficient; 95% CI: 95% confidence interval of the mean.

**Table 2:** Biomechanical parameters derived from corneal visualization Scheimpflug technology by gender and mean ± standard deviation (range).

Parameters	All (*n* = 783)	Female (*n* = 402)	Male (*n* = 381)	*P* value
First applanation time (ms)	7.33 ± 0.32 (6.63~8.39)	7.32 ± 0.29 (6.7~8.39)	7.35 ± 0.34 (6.63~8.38)	0.667^†^
First applanation length (mm)	1.79 ± 0.07 (1.38~1.9)	1.79 ± 0.05 (1.43~1.9)	1.78 ± 0.08 (1.38~1.9)	0.940^†^
Velocity in (m/s)	0.15 ± 0.02 (0.11~0.2)	0.15 ± 0.01 (0.11~0.19)	0.15 ± 0.02 (0.11~0.2)	0.370^*∗*^
Second applanation time (ms)	22.09 ± 0.45 (20.81~23.52)	22.07 ± 0.44 (20.81~23.52)	22.11 ± 0.45 (21.07~23.39)	0.218^†^
Second applanation length (mm)	1.68 ± 0.32 (0.84~2.29)	1.69 ± 0.32 (0.89~2.15)	1.67 ± 0.32 (0.84~2.29)	0.360^†^
Velocity out (m/s)	−0.39 ± 0.07 (−0.68~−0.17)	−0.39 ± 0.07 (−0.61~−0.17)	−0.39 ± 0.07 (−0.68~−0.19)	0.935^*∗*^
Highest concavity time (ms)	16.72 ± 0.49 (14.78~18.71)	16.7 ± 0.5 (14.78~18.25)	16.74 ± 0.49 (15.25~18.71)	0.258^†^
Deformation amplitude (mm)	1.07 ± 0.09 (0.84~1.29)	1.06 ± 0.08 (0.85~1.27)	1.07 ± 0.09 (0.84~1.29)	0.257^*∗*^
Peak distance (mm)	5.01 ± 0.24 (4.18~5.64)	5.01 ± 0.23 (4.4~5.59)	5.02 ± 0.25 (4.18~5.64)	0.657^*∗*^
Highest concavity radius (mm)	6.93 ± 0.79 (4.67~9.43)	6.89 ± 0.8 (4.93~9.43)	6.96 ± 0.78 (4.67~9.39)	0.177^*∗*^

^*∗*^Student's two-sample *t*-test.

^†^Wilcoxon rank-sum test.

**Table 3:** Linear regression analyses on velocity in, velocity out, deformation amplitude, peak distance, and highest concavity radius.

Parameter	Velocity in (m/s)				Velocity out (m/s)				Deformation amplitude (mm)				Peak distance (mm)				Highest concavity radius (mm)			
	Univariate model		Multivariate model^*∗*^		Univariate model		Multivariate model^*∗*^		Univariate model		Multivariate model^*∗*^		Univariate model		Multivariate model^*∗*^		Univariate model		Multivariate model^*∗*^	
	*β* value	*P* value	*β* value	*P* value	*β* value	*P* value	*β* value	*P* value	*β* value	*P* value	*β* value	*P* value	*β* value	*P* value	*β* value	*P* value	*β* value	*P* value	*β* value	*P* value
Age	−0.016	0.662	—	—	0.005	0.881	—	—	−0.426	0.67	—	—	−0.029	0.421	—	—	−0.021	0.561	—	—
Gender, female	0.032	0.37	—	—	−0.003	0.935	—	—	−0.041	0.257	—	—	−0.016	0.657	—	—	−0.048	0.177	—	—
Intraocular pressure	−0.332	<0.001	−0.318	<0.001	0.536	<0.001	0.46	<0.001	−0.521	<0.001	−0.503	<0.001	−0.578	<0.001	−0.504	<0.001	0.378	<0.001	0.298	<0.001
Central corneal thickness	−0.268	<0.001	−0.148	<0.001	0.353	<0.001	0.197	<0.001	−0.276	<0.001	−0.115	<0.001	−0.244	<0.001	−0.088	0.002	0.344	<0.001	0.248	<0.001
Mean keratometry	0.237	<0.001	0.267	<0.001	0.011	0.754	—	—	0.035	0.328	0.08	0.009	−0.0313	<0.001	−0.239	<0.001	−0.02	0.578	—	—
Corneal volume	−0.107	0.003	—	—	0.253	<0.001	—	—	−0.25	<0.001	—	—	−0.289	<0.001	—	—	0.219	<0.001	—	—
Anterior chamber depth	−0.018	0.623	—	—	−0.196	<0.001	−0.141	<0.001	−0.049	0.17	−0.095	0.002	0.245	<0.001	0.167	<0.001	−0.096	0.007	—	—
Anterior chamber volume	−0.039	0.275	—	—	−0.225	<0.001	—	—	−0.04	0.265	—	—	0.297	<0.001	—	—	−0.13	<0.001	—	—

^*∗*^Adjusted for age, gender, intraocular pressure, central corneal thickness, mean keratometry, and anterior chamber depth; adjusted *R* ^2^ = 0.205 for velocity in, 0.341 for velocity out, 0.298 for deformation amplitude, 0.425 for peak distance, and 0.196 for highest concavity radius.

## Abbreviations

A1-time: First applanation time, time from the initiation of the air puff until the first applanation
A1-length: First applanation length, length of the flattened cornea at the first applanation
*V*_in_: Velocity in, corneal velocity during the first applanation
HC-time: Highest concavity time, time from the start until highest concavity of the cornea is reached
HC-radius: Highest concavity-radius, radius of curvature at the time of highest concavity
PD: Highest concavity peak distance, distance of the two surrounding “knees” at highest concavity
DA: Highest concavity deformation amplitude, deformation amplitude from start to highest concavity at the corneal apex
A2-time: Second applanation time, time from the initiation of the air puff until the second applanation
A2-length: Second applanation length, length of the flattened cornea at the second applanation
*V*_out_: Velocity out, corneal velocity during the second applanation.
